# Genetic and genomic analyses underpin the feasibility of concomitant genetic improvement of milk yield and mastitis resistance in dairy sheep

**DOI:** 10.1371/journal.pone.0214346

**Published:** 2019-11-25

**Authors:** Georgios Banos, Emily L. Clark, Stephen J. Bush, Prasun Dutta, Georgios Bramis, Georgios Arsenos, David A. Hume, Androniki Psifidi

**Affiliations:** 1 Scotland’s Rural College, Edinburgh, Easter Bush, Midlothian, Scotland, United Kingdom; 2 The Roslin Institute, University of Edinburgh, Easter Bush, Midlothian, Scotland, United Kingdom; 3 School of Veterinary Medicine, Aristotle University of Thessaloniki, Thessaloniki, Greece; 4 Nuffield Department of Clinical Medicine, University of Oxford, John Radcliffe Hospital, Headington, Oxford, England, United Kingdom; 5 Mater Research Institute-University of Queensland, Translational Research Institute, Woolloongabba, Australia; 6 Royal Veterinary College, University of London, Hatfield, England, United Kingdom; University of Florida, UNITED STATES

## Abstract

Milk yield is the most important dairy sheep trait and constitutes the key genetic improvement goal via selective breeding. Mastitis is one of the most prevalent diseases, significantly impacting on animal welfare, milk yield and quality, while incurring substantial costs. Our objectives were to determine the feasibility of a concomitant genetic improvement programme for enhanced milk production and resistance to mastitis. Individual records for milk yield, and four mastitis-related traits (milk somatic cell count, California Mastitis Test score, total viable bacterial count in milk and clinical mastitis presence) were collected monthly throughout lactation for 609 ewes of the Chios breed. All ewes were genotyped with a mastitis specific custom-made 960 single nucleotide polymorphism (SNP) array. We performed targeted genomic association studies, (co)variance component estimation and pathway enrichment analysis, and characterised gene expression levels and the extent of allelic expression imbalance. Presence of heritable variation for milk yield was confirmed. There was no significant genetic correlation between milk yield and mastitis traits. Environmental factors appeared to favour both milk production and udder health. There were no overlapping of SNPs associated with mastitis resistance and milk yield in Chios sheep. Furthermore, four distinct Quantitative Trait Loci (QTLs) affecting milk yield were detected on chromosomes 2, 12, 16 and 19, in locations other than those previously identified to affect mastitis resistance. Five genes (*DNAJA1*, *GHR*, *LYPLA1*, *NUP35* and *OXCT1*) located within the QTL regions were highly expressed in both the mammary gland and milk transcriptome, suggesting involvement in milk synthesis and production. Furthermore, the expression of two of these genes (*NUP35* and *OXCT1*) was enriched in immune tissues implying a potentially pleiotropic effect or likely role in milk production during udder infection, which needs to be further elucidated in future studies. In conclusion, the absence of genetic antagonism between milk yield and mastitis resistance suggests that simultaneous genetic improvement of both traits be achievable.

## Introduction

The world’s commercial dairy sheep industry is primarily concentrated in Mediterranean countries and linked to local breeds; milk is mostly used to produce high quality cheeses and other dairy products. Milk yield represents more than two thirds of the total income of the dairy sheep sector [1] and, therefore, increasing milk yield is the most important and sometimes only objective of selective breeding. Milk production traits in dairy sheep are lowly to moderately heritable, with reported heritability estimates ranging from 0.13 to 0.51 [2, 3] and amenable to improvement with traditional selective breeding programmes based on pedigree and phenotypic data. Indeed, such programmes have been established in many sheep populations over recent decades [2, 4]. Incorporation of genomic information in some breeding programmes (e.g. French Lacaune, Spanish Churra, Italian Sarda) has led to an acceleration of the genetic improvement outcomes.

The Greek Chios breed is considered to be among the most productive and prolific dairy sheep breeds worldwide [5]. A traditional breeding programme for the enhancement of milk yield has been in place since year 2000 for this breed, leading to substantial improvement in this trait. However, further increases in milk yield may be achieved with the use of relevant genomic information.

Beyond simply increasing milk production, the dairy sheep industry faces challenges such as the need to offer healthy products to consumers, addressing animal welfare, and ensuring the long-term competitiveness and sustainability of the sector. Mastitis is the most prevalent and costly disease in the dairy industry due to reduced and discarded milk, early involuntary culling of animals, and veterinary services and labour costs [6, 7]. The disease also poses a potential threat of zoonosis and antimicrobial resistance if antibiotic treatment is not applied carefully [6–8]. Moreover, mastitis is a welfare concern because of associated pain, anxiety and restlessness, and upsets the normal feeding behaviour of the animals [9]. Host resistance to mastitis is generally a lowly heritable trait, with heritability estimates previously reported ranging from 0.10 to 0.20 [7]. Recently, an ovine custom made mastitis specific 960-SNP DNA array was built to facilitate genetic selection and improvement of animal resistance to mastitis in dairy sheep [10] [11] [12] [13]. We previously used this array in a targeted genomic association study and detected five quantitative trait loci (QTLs) for mastitis resistance in Chios sheep [10].

In the present study, we examined the genetic relationship between milk yield and mastitis resistance in the Chios sheep, using pedigree and genomic information. Mastitis resistance was manifested with four relevant measured traits, namely milk somatic cell count, California Mastitis Test score, total viable bacterial count in milk and clinical manifestation of the disease. The relationship between milk yield and these -mastitis traits is crucial if enhancing mastitis resistance is to be included in the selective breeding goal together with increasing milk production. We estimated genetic parameters and investigated whether SNP markers previously found to be associated with mastitis resistance in Chios sheep were also associated with milk yield, using the ovine custom-made mastitis-specific array. We also performed pathway analysis and examined gene expression and allelic expression imbalance to assess whether genes located under the QTL regions, were enriched in tissues relevant to milk yield and mastitis resistance.

## Materials and methods

### Ethical statement

The study was approved by the Ethics and Research Committee of the Faculty of Veterinary Medicine, Aristotle University of Thessaloniki, Greece. Permits for access and use of the commercial farms were granted by the farm owners, who were members of the Chios Sheep Breeders’ Cooperative “Macedonia”. During sampling, animals were handled by qualified veterinarians. Permission to qualified veterinarians to perform milk and blood sampling was granted by the National (Greek) Legislature for the Veterinary Profession, No. 344/29-12-2000.

### Animals, sampling and phenotyping

Animals used in the present study included 609 purebred Chios dairy ewes raised in four commercial farms in Greece. Complete pedigree data were available comprising a total of 38,459 animals, 1,892 sires and 20,634 dams. Ewes were in their first or second lactation. Daily milk yield was recorded on each animal on the day of monthly visits to the farms during the first five months of lactation. The first milk yield record was obtained at least three days after lamb weaning (ca. 42 days post lambing), which signals the onset of lactation. The total number of individual animal records was 2,436. Animal records for clinical mastitis occurrence (CM) and three mastitis indicator traits (milk somatic cell count (SCC), California Mastitis Test (CMT) score and total viable bacterial count (TVC) in milk) were also collected at the time of these visits by a qualified veterinarian. On the day of visit, the presence or absence (0/1) of CM was recorded and two 50 ml milk samples were collected in the milking parlour under aseptic conditions for the measurement of CMT, SCC and TVC. CMT was scored on a scale from 0 to 4, with high values indicating the presence of elevated SCC and, potentially, pathogens in milk; this test was performed with a commercial kit according to manufacturer’s instructions (Bovi-vet, Kruuse, Germany). SCC was measured with Fossomatic 360 (Foss Electric, Hillerød, Denmark) and expressed as the number of cells/ml of milk. TVC was measured with Bactoscan FC 50 (Foss Electric, Hillerød, Denmark) and expressed as the number of viable bacteria/ml of milk. The three mastitis indicator traits, CMT, SCC and TVC, may capture subclinical mastitis incidences and reflect the general health status of the udder. Peripheral blood samples were taken from each ewe in 9 ml K_2_EDTA Vacutainer blood collection tubes (BD diagnostics) by jugular venepuncture for genomic DNA extraction.

### Genetic parameter estimation

Genetic parameters for milk yield were estimated using the following basic mixed model:

$$Y_{ijkmno}=\mu +F_{i}+YS_{j}+a_{1}\cdot age+L_{k}+\sum_{n=1}^{2}b_{n}P_{n}W_{m}+g_{o}+pe_{o}+e_{ijkmno}$$ (1)

Where: Y = record of ewe *o* in week of lactation *m*

*μ* = overall mean

*F* = fixed effect of flock (farm) *i*

*YS* = fixed effect of year-season of lambing *j*

*α*_1_ = linear regression on age at lambing (*age*)

*L* = fixed effect of lactation number *k*

*W* = fixed effect of week of lactation (i.e. week post-lambing) *m*

*b*_*n*_ = fixed regression coefficient on week of lactation *m* (order n = 2)

*P*_*n*_ = orthogonal polynomial of week *m* (order n = 2)

*g* = random additive genetic effect of ewe *o*, including pedigree genetic relationships among animals

*pe* = random permanent environment effect of ewe *o*

*e* = random residual effect

Heritability and repeatability estimates were derived from the variance components calculated for the random effects in model (1). In a separate analysis, the additive genetic and permanent environment effects in model (1) were replaced by interactions of the latter with second-order polynomial functions of week of lactation. The choice of polynomial order was decided after testing sequentially increasing orders with the log-likelihood test. This analysis resulted in distinct variance component and genetic parameter estimates by week of lactation, which were then combined to derive average heritability and repeatability estimates for early (weeks of lactation 1–7), mid (weeks 8–17) and late (weeks 18–24) lactation. In addition, genetic correlations between milk yields measured at different lactation stages were calculated based on corresponding genetic covariance estimates. A smoothed lactation curve adjusted for all fixed effects in the model was also derived.

Finally, bivariate analyses of milk yield and each one of the four mastitis related traits were conducted using model (1). The four mastitis traits were analysed as described in [10]. Briefly, SCC and TVC data, which were originally significantly skewed, were log-transformed to ensure a normal distribution. CM was recorded as a 0/1 trait and, therefore, a logit function was fitted to account for its binary nature. Outcomes from the bivariate analyses were used to estimate phenotypic and genetic correlations between traits.

All statistical analyses in the present study were conducted with ASReml v4.0 [14].

### Targeted genomic association analysis

DNA was extracted from blood buffy coat as described previously [15].

All animals were genotyped with a customised mastitis specific 960 SNP DNA array containing SNPs located on chromosomes 2, 3, 5, 12, 16 and 19. Briefly, this array was built based on QTLs for mastitis resistance found to segregate in multiple different dairy sheep breeds. For the design of this custom-made array, SNPs were selected from both 50K and 800K SNP ovine DNA arrays, as well as from re-sequencing data. The average density of the array was 1 SNP every 23 Kb (for more details see [10]). This genomic tool was built within an FP7 European research project (http://cordis.europa.eu/result/rcn/163471_en.html). Genotypes at each SNP locus were subjected to quality control measures using PLINK v1.9[16]. After excluding SNPs with minor allele frequency < 0.05 and/or call rate < 0.95%, 731 SNP markers remained for further analysis.

Possible population stratification was investigated with the use of the genomic relationship matrix among individual animals. This matrix was converted to a distance matrix that was then used to conduct multidimensional scaling analysis using the R package GenABEL v1.8[17].

Individual ewe phenotypes were residuals resulted after fitting a model that included all fixed effects of model (1); thus, phenotypic records were adjusted for all these environmental effects. Separate phenotypes were derived for the entire lactation (overall) and for each lactation stage (early, mid, late) as described above. In all cases, GEMMA v0.94.1 [18] was used to conduct genomic association analyses based on a mixed model that included the genomic relationship matrix among individual ewes as a polygenic effect. After Bonferroni correction for multiple testing, the significance threshold for nominal *P<*0.05 was set at *P<*6.83x10^-5^ and a suggestive threshold (accounting for one false positive per genome scan) was set at *P<*1.36x10^-3^.

Statistically significant SNPs from the genomic association analyses were further examined with a mixed model that included the fixed effects of model (1), the fixed effect of the SNP genotype and the random effect of the animal including the pedigree relationship matrix. Additive (a) and dominance (d) effects were calculated as follows:

a = (AA-BB)/2
d = AB-((AA+BB)/2)

where AA, BB and AB were the marginal means of the respective genotype. All analyses were conducted with ASReml v4.0 [14].

Linkage disequilibrium (LD) among significant SNPs was calculated based on the *r*^*2*^ value using PLINK v1.9 [16]. Blocks of LD in regions harbouring significant SNPs were visualised using Haploview v4.2 [19].

All significant (post-Bonferroni correction) and suggestive SNPs identified in the genomic analysis for milk yield were mapped to the reference genome and annotated using the Ensembl variant effect predictor (http://www.ensembl.org/Tools/VEP) tool and the Oar v3.1 assembly. Moreover, annotations for genes located both up- and down-stream (0.2 Mb) of the significant markers in the candidate regions for milk yield were obtained from Ensembl BioMart data mining tool (http://www.ensembl.org/biomart/martview/) and the Oar v3.1 assembly.

### Pathway analysis

The list of annotated genes located within the QTL regions for milk yield identified in the present study were analysed with the Ingenuity Pathway Analysis (IPA) programme (www.ingenuity.com) in order to identify canonical pathways and gene networks constructed by the products of these genes. All genes located in the genomic regions targeted by the custom-made DNA array used in our study constituted the background of this analysis. IPA constructs multiple possible upstream regulators, pathways and networks which may be associated with the biological mechanism underlying the studied trait. The analysis is based on data from large-scale causal networks derived from the Ingenuity Knowledge Base. IPA then infers the most suitable pathways and networks based on their statistical significance, after correcting for a baseline threshold [20]. The IPA score in the constructed networks can be used to rank these networks based on the P-values obtained using Fisher’s exact test (IPA score or P*-*score = –log_10_(P value)).

### Gene expression analysis

We performed gene expression analyses to assess whether genes located within the candidate regions for milk yield were enriched in tissues relevant to milk yield and/or mastitis, assuming enrichment indicated functional relevance. Genes contributing to milk production are likely to be expressed in milk somatic cells, mammary gland, and other organs such as the liver and kidney that provide nutrients and regulate the electrolytes needed for lactosynthesis and the production of milk. We also reasoned that the expression of genes with pleiotropic effects would be associated with both milk yield and resistance to mastitis, and/or expressed in both mammary gland and immune related tissues. To assess the expression profiles of genes located in the candidate regions for milk yield, we obtained publicly available data from an RNA-seq characterisation of the milk transcriptome of two Spanish dairy sheep breeds, Churra and Assaf, where milk somatic cells of eight individual sheep (four from each breed) had been sampled throughout lactation at 10, 50, 120 and 150 days after lambing [21, 22]. To supplement this data, we used publicly available RNA-Seq data from a high-resolution atlas of gene expression across tissues and cell types from all major organ systems in sheep [23, 24]. The sheep gene expression atlas, which includes 437 RNA-Seq libraries was produced using six Texel x Scottish Blackface sheep [23]. An additional 83 RNA-Seq libraries from a Texel trio (ewe, lamb and ram) were included in the sheep gene expression atlas [24]. We extracted data pertaining to the mammary gland, liver and kidney. Since we were interested in detecting genes related to both milk yield and mastitis, we also extracted the expression level of the genes under consideration in immune-related tissues, specifically hemolymph nodes, mesenteric, popliteal, prescapular and submandibular lymph nodes, peripheral blood mononuclear cells, blood leukocytes, monocyte-derived macrophages, bone marrow derived macrophages, alveolar macrophages, and tonsils.

Expression levels for all samples, were estimated using Kallisto v0.42.4 [25]. To reduce batch effects when combining data from different sources, particularly those employing different RNA selection methods [26], expression was quantified using a set of transcripts constituting a standardised transcriptomic space, as described in [27] and [28]. Expression was reported for each protein-coding transcript as the number of transcripts per million, and then summarised to the gene-level (as in [29]). Heatmaps were drawn using the heatmap.2 function of the R package gplots v3.0.1, in order to demonstrate expression enrichment in the different tissues and lactation stages.

### Variant calling and allelic expression imbalance analysis

Much of the genetic variation in genes that control a quantitative trait is likely to affect their transcriptional regulation. In fact, many quantitative traits associated with altered gene expression, and trait-associated loci are enriched for eQTLs [30]. If an individual is heterozygous for a *cis*-acting mutation it is expected that the two alleles of the gene will be expressed unequally causing allelic expression imbalance. Measuring the relative expression levels of two alleles using RNA-Seq may lead to the identification of *cis*-acting SNPs or haplotypes [31–34]. To identify any *cis*-QTLs affecting the genes located in the candidate regions for milk yield we obtained the raw RNA-Seq data for mammary gland tissue from three adult female Texel x Scottish Blackface sheep from the sheep gene expression atlas [23]. The aligner HISAT2 (v2.0.4) [35], was used to produce the BAM files as previously described [23]. Variants were called using BCFtools [36] mpileup (v1.4) with parameters—max-depth 1000000—min-MQ 60, followed by BCFtools call (v1.4) with parameters -m (allow multiallelic variants) and -v (variant only). The minimum MAPQ (mapping quality) score was chosen to focus on uniquely mapped reads for variant calling. The resulting VCF file contained both SNPs and indels. The exonic variants of the protein coding genes located in the milk yield candidate regions were obtained from each VCF file using the program GTF_Extract (v0.9.1) (https://github.com/fls-bioinformatics-core/GFFUtils/blob/master/docs/GTF_extract.rst) and BEDtools [37] intersect (v2.25.0) based on gene annotations from Ovis_aries.Oar_v3.1. The putative functional impact of each variant on the encoded proteins was predicted using SnpEff v4.3 [38] with the parameter–onlyProtein (only annotate protein-coding variants). BCFtools norm (v1.4) with parameter–d was used to remove duplicated VCF records that arose due to duplicated exon coordinates in the GTF file (that is, exons present in more than one transcript). Finally, VCFs from each animal were filtered to obtain only biallelic heterozygous SNPs, using BCFtools ‘view’ (v1.4). For the allelic expression imbalance analysis we focused on biallelic heterozygous exonic SNPs, since the non-exonic variants may signify transcriptional noise in mRNA sequencing and contribute potential errors in the analysis.

Read counts for both the reference and alternate allele were obtained using allelecounter v0.6 (https://github.com/secastel/allelecounter) with parameters—min_cov 4,—min_baseq 20 and—min_mapq 60 and—max_depth 10000. Allelic expression imbalance, per gene, was estimated using MBASED (Meta-analysis Based Allele-Specific Expression Detection) [39] with parameters isPhased = FALSE, numSim = 10^6, BPPARAM = SerialParam(). MBASED allelic expression imbalance estimates were derived by combining information across individual heterozygous SNP within a gene. Only variants with >10 reads in either reference or alternate allele were used. We retained only those genes with Benjamin-Hochberg [40] adjusted *P* <0.05 and major allele frequency > 0.7.

## Results

### Descriptive statistics

An average daily milk yield of 1,912 grams (g) was produced in the studied sheep population with a standard deviation of 713 g, a maximum of 4,597 g and a minimum of 210 g. As expected, milk yield decreased as lactation progressed [41].

All fixed effects that were included in the model of statistical analysis accounted for a significant (*P<*0.05) proportion in milk variation. This can be exemplified by the average daily milk yield ranging from 1,787 g in lactation 1 (368 ewes) to 2,134 g in lactation 2 (241 ewes). Including these significant sources of systematic variation in the model as fixed effects ensured the unbiasedness of the variance component estimates of the random effects and corresponding genetic parameters presented next.

### Genetic parameters

Estimates of heritability and repeatability of milk yield (Table 1) were derived for the entire lactation as well as different stages of lactation defined as early, mid and late. Statistically significant (*P<*0.05) moderate trait heritabilities (0.19–0.28) and repeatabilities (0.69–0.76) were estimated across all lactation stages. Moreover, the genetic correlations between milk yield in different lactation stages were significantly (*P*<0.05) positive. Specifically, average genetic correlation between milk production in early and mid lactation was 0.89, early and late lactation 0.60, and mid and late lactation 0.86. The genetic correlation between early and late lactation was significantly (*P<*0.05) lower than unity. In practical terms, lactation onset, peak lactation and lactation persistence may have partly separate genetic control.

**Table 1:** Heritability (h^2^) and repeatability (r) estimates of daily milk yield in Chios sheep by lactation stage and across the entire lactation; standard errors in parentheses.

Parameter	Early lactation (1–7 weeks)	Mid lactation (8–17 weeks)	Late lactation (18–24 weeks)	Overall lactation
**h**^**2**^	0.28 (0.06)	0.19 (0.06)	0.23 (0.06)	0.23 (0.06)
**r**	0.76 (0.02)	0.69 (0.02)	0.71 (0.02)	0.71 (0.02)

Genetic correlations between milk and mastitis traits measured throughout the entire lactation were not significantly different from zero (*P*>0.05). Negative phenotypic correlations were observed between these traits (*P*<0.05), indicative of favourable environmental effects to both production and health (Table 2).

**Table 2:** Estimates of phenotypic and genetic correlations between milk yield and four mastitis traits measured throughout the entire lactation in Chios sheep; standard errors in parentheses.

Mastitis trait	Phenotypic correlation	Genetic correlation
**SCC**	-0.18 (0.04)*	-0.12 (0.14)
**CMT**	-0.18 (0.04)*	-0.12 (0.13)
**TVC**	-0.10 (0.03)*	-0.11 (0.14)
**CM**	-0.07 (0.04)	-0.09 (0.19)

SCC: milk somatic cell count, CMT: California Mastitis Test score, TVC: total bacterial count in milk, CM: clinical mastitis occurrence

*Significantly different from zero (*P<*0.05)

### Targeted genomic association analysis

**Fig 1:**
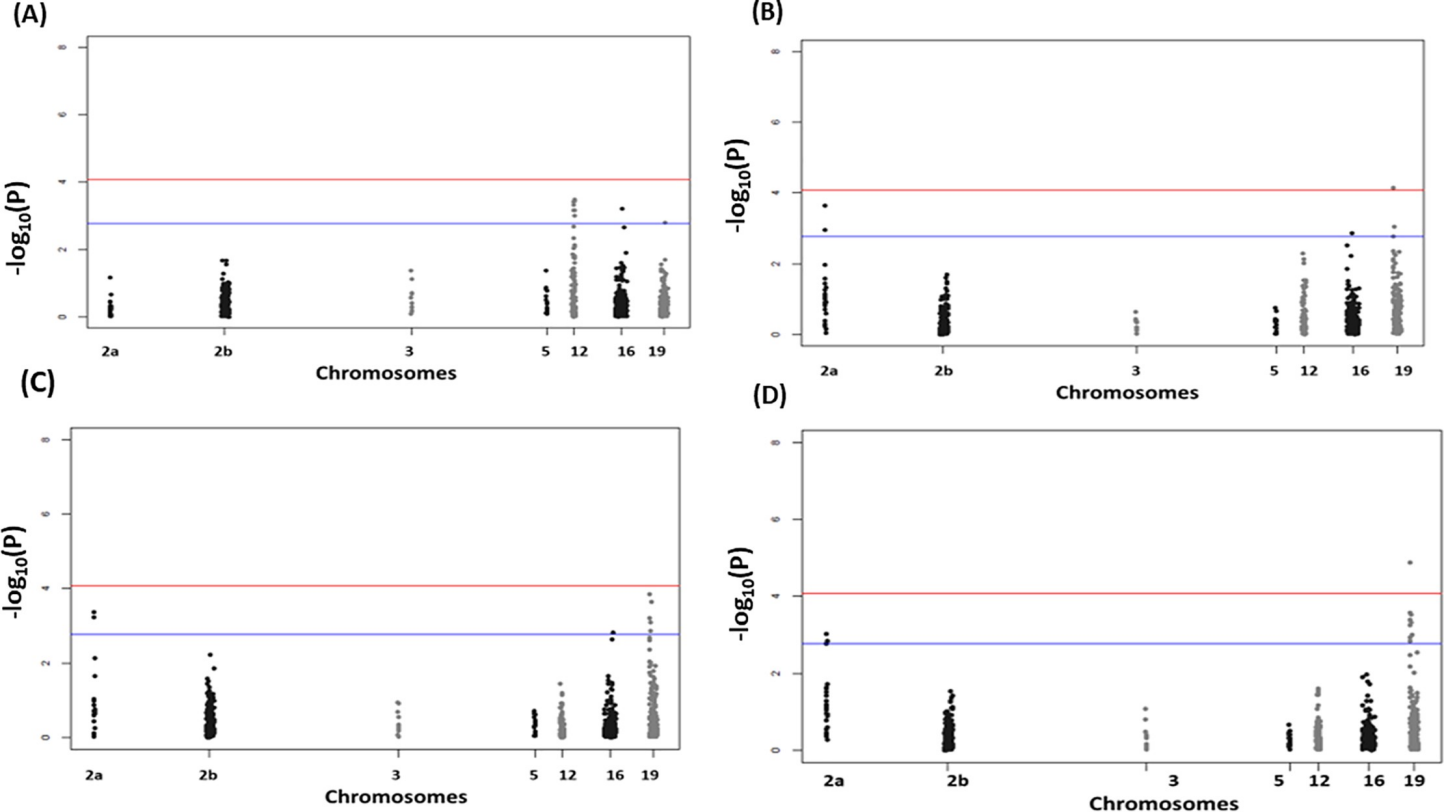
Manhattan plots displaying the genomic association results for milk yield in Chios sheep. Manhattan plots for milk yield in early (A), mid (B), late (C), and overall (D) lactation. Genomic location is plotted against -log_10_(*P*). Red and blue lines, respectively, are thresholds for significance post-Bonferroni correction (*P*<0.05) and for suggestive significance (accounting for one false positive per genome scan).

**Fig 2:**
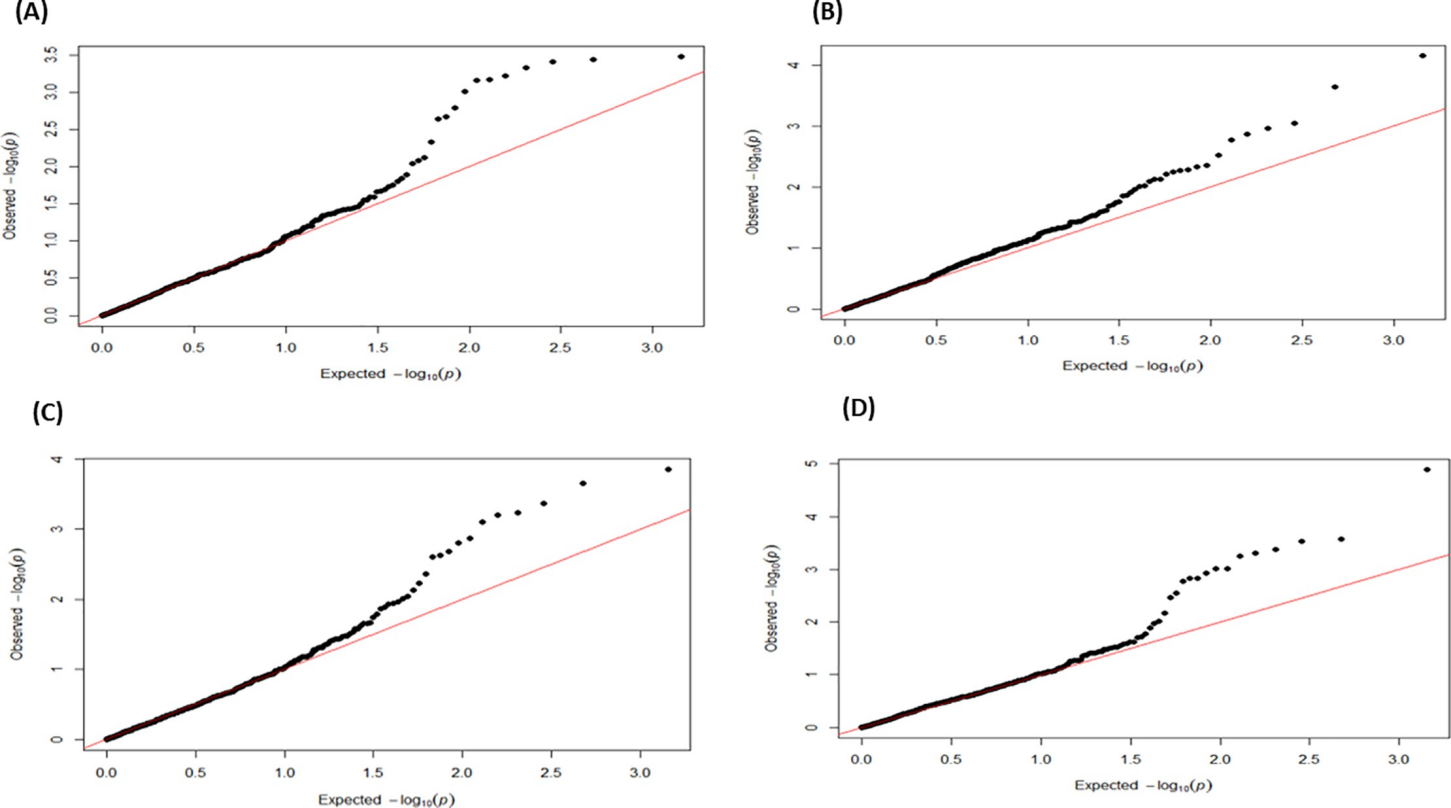
Q-Q plots displaying the genomic association results for milk yield in Chios sheep. Q-Q plots in early (A), mid (B), late (C) and overall (D) lactation; observed *P-*values are plotted against the expected *P-*values.

Separate targeted genomic association analyses were conducted for milk yield in early, mid, late and overall lactation. Multidimensional scaling analysis of the studied population revealed no substructure. In general, similar genomic associations were detected for milk yield in middle, late and overall lactation but distinct associations were observed in early lactation. We identified a statistically significant association after Bonferroni correction for multiple testing on chromosome 19 (*P* = 1.28 x 10^−5^) and three suggestive associations on chromosomes 2 (*P* = 2.27 x 10^−4^), 12 (*P* = 3.35 x 10^−4^) and 16 (*P* = 6.03 x 10−4). Details of SNPs associated with milk yield are shown in Table 3. Manhattan plots and corresponding Q-Q plots displaying genomic association results are shown in Fig 1 and Fig 2, respectively.

**Table 3:** List of Single Nucleotide Polymorphisms (SNPs) associated with milk yield in Chios sheep.

Lactation stage	SNP	Chr (position)	P-value	Add(P-value)	Dom(P-value)	p	q
**Early** **1–7 weeks**	OAR12_23075585	12(20050780)	3.35E-04	0.07(0.09)	0.07(0.10)	0.62	0.38
	oar3_OAR12_19689222	12(19689222)	3.65E-04	0.05(0.36)	0.12(0.03)	0.73	0.27
	oar3_OAR12_19269103	12(19269103)	4.66E-04	-0.03(0.33)	0.05(0.02)	0.73	0.27
	oar3_OAR12_19500329	12(19500329)	6.98E-04	0.08(0.02)	0.02(0.64)	0.6	0.4
	oar3_OAR12_19624437	12(19624437)	6.77E-04	0.05(0.30)	0.09(0.10)	0.72	0.28
	oar3_OAR12_19840123	12(19840123)	9.80E-04	0.04(0.30)	0.08(0.10)	0.68	0.32
	oar3_OAR16_33078067	16(33078067)	6.03E-04	-0.09(0.00)	0.20(0.05)	0.96	0.04
**Middle** **8–17 weeks**	OAR19_25259444	19(23804520)	7.03E-05	-0.15 (0.00)	0.05 (0.25)	0.48	0.52
	oar3_OAR19_24119431	19(24119431)	9.03E-04	-0.14(0.00)	-0.02(0.53)	0.48	0.52
	OAR19_25513179	19(24010793)	1.70E-03	-0.15(0.00)	-0.01(0.77)	0.58	0.42
	OAR16_34906481	16(32156238)	1.29E-03	-0.08(0.02)	0.07(0.05)	0.90	0.10
	OAR2_133418483	2(125230366)	1.45E-03	0.08(0.51)	-0.10(0.44)	0.92	0.08
	OAR2_133088440	2(124907852)	-2.27E-04	0.23(0.00)	0.19(0.03)	0.82	0.18
	oar3_OAR2_124936445	2(124936445)	1.11E-03	0.12(0.06)	0.07(0.30)	0.78	0.22
**Late** **18–24 weeks**	OAR19_25259444	19(23804520)	1.38E-04	-0.15(0.00)	-0.02(0.57)	0.48	0.52
	oar3_OAR19_24745933	19(24745933)	2.17E-04	0.10(0.00)	-0.10(0.00)	0.54	0.46
	OAR19_25830151	19(24342061)	1.35E-03	0.07(0.07)	-0.00(0.87)	0.72	0.28
	oar3_OAR19_24707843	19(24707843)	7.83E-04	-0.13(0.00)	-0.09(0.03)	0.64	0.36
	oar3_OAR19_23656789	19(23656789)	6.25E-04	-0.11(0.00)	-0.06(0.11)	0.52	0.48
	oar3_OAR2_124936445	2(124936445)	5.53E-04	0.11(0.02)	-0.00(0.93)	0.78	0.22
	OAR2_133088440	2(124907852)	4.15E-04	0.19(0.00)	0.09(0.18)	0.82	0.18
**Overall**	OAR19_25259444	19(23804520)	1.28E-05	-0.14(0.00)	-0.00(0.84)	0.48	0.52
	oar3_OAR19_24032312	19(24032312)	2.70E-04	-0.11(0.00)	-0.08(0.03)	0.6	0.4
	oar3_OAR19_24707843	19(24707843)	2.90E-04	-0.12(0.00)	-0.07(0.04)	0.64	0.36
	OAR19_25513179	19(24010793)	4.16E-04	-0.11(0.00)	-0.06(0.07)	0.58	0.42
	oar3_OAR19_24119431	19(24119431)	4.79E-04	-0.11(0.00)	-0.09(0.01)	0.48	0.52
	oar3_OAR19_23929524	19(23929524)	5.62E-04	0.11(0.00)	-0.03(0.29)	0.48	0.52
	oar3_OAR19_24745933	19(24745933)	9.35E-04	0.10(0.00)	-0.05(0.15)	0.54	0.46
	oar3_OAR19_23891277	19(23891277)	1.17E-03	-0.10(0.00)	-0.05(0.13)	0.54	0.46
	oar3_OAR19_23656789	19(23656789)	1.44E-03	-0.10(0.00)	-0.05(0.15)	0.52	0.48
	OAR2_133088440	2(124907852)	9.47E-04	0.20(0.00)	0.12(0.06)	0.82	0.18
	OAR2_133418483	2(125230366)	1.44E-03	0.10(0.30)	-0.08(0.43)	0.92	0.08

P–value: P-value from genomic association study; additive allele substitution effect (ADD) and corresponding P-value; dominance effect (DOM) and corresponding P-value; p and q allelic frequencies; SNP position is based on Oar v3.1 assembly.

The significance of the above SNP markers was confirmed in mixed model analyses based on the pedigree genetic relationship matrix. The additive and dominance genetic effects of each SNP in the corresponding lactation stage, are summarised in Table 3. Most SNPs had a significant additive effect and a few a significant dominance effect on milk yield. When located on the same chromosomes, the significant markers identified for milk yield were in linkage disequilibrium (LD measured as r^2^ = 0.27–0.97), implying that they correspond to the same causative mutation (S1 Table). Most importantly, the significant SNPs identified in the present study were not in LD with the SNPs previously associated with the mastitis related traits in Chios sheep [10] (S1 Table). Only small (less than 200kb length) LD blocks were visualised with Haploview, probably due to a high number of recombination events having taken place in the studied outbred population. All significant SNP markers were located in intergenic or intronic regions. The candidate QTL regions for milk yield contained a relatively small number of protein-coding genes (n = 13), microRNAs (n = 3) and non-coding RNAs (n = 3) (S2 Table).

### Pathway analysis

**Fig 3:**
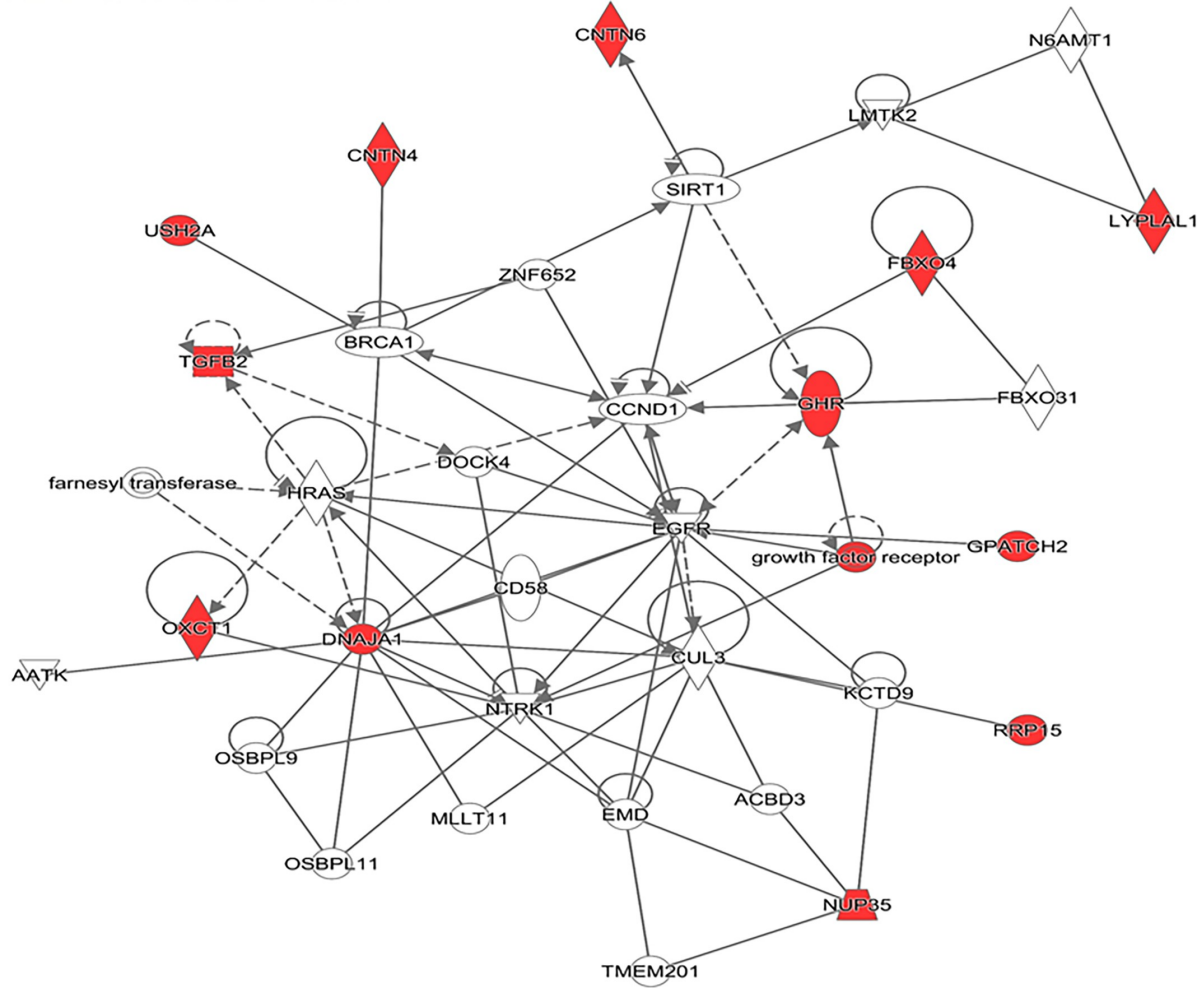
Network analysis using the IPA software. A gene network related to organ, organismal and embryonic development. The network illustrates the molecular interactions between candidate gene products for milk yield. Arrows with solid lines represent direct interactions and arrows with broken lines represent indirect interactions. Genes with white labels are those added to the IPA analysis because of their interaction with the target gene products (in red).

One significant (IPA score = 34) network of molecular interactions related with organ development, organismal development and embryonic development was constructed from the genes located in the candidate regions for milk yield (Fig 3).

### Gene expression analysis

Six of the genes located in the candidate regions for milk yield (*DNAJA1*, *GHR*, *LYPLAL1*, *NUP35*, *OXCT1*and *RRP15)* were expressed in either the milk transcriptome or the mammary gland (S1–S3 Figs). The growth hormone receptor (*GHR*) and 3-oxoacid CoA transferase 1 (*OXCT1*) genes were highly expressed in liver and kidney cortex tissue, respectively (S2 Fig). Moreover, *OXCT1* and *NUP35*, detected in tissues related to milk production, were also enriched in immune related tissues, relative to the other tissues analysed (S3 Fig).

### Allelic expression imbalance analysis

Exonic SNP and indels were observed in all the protein coding genes located in the candidate regions for milk yield. Missense variants were identified in several genes including *CNTN4*, *DNAJC1*, *GHR*, *NUP35* and *RRP15*. One-sampled MBASED analysis identified only one gene *RRP15* (*P = 3e-03*) with significant allelic expression imbalance. Specifically, two synonymous SNPs in *RRP15* (major allele frequency 0.71) were detected exhibiting allelic expression imbalance (S3 Table). However, these results should be interpreted with caution since allelic expression imbalance was evident in only one of the three individual sheep.

### Candidate genes

Based on all above results, a total of four genes (*DNAJA1*, *GHR*, *LYPLA1* and *OXCT1*) were selected as putative candidate genes for milk yield in Chios sheep (S4 Table). Genes were selected using a combination of their known biological function, involvement in relevant networks, enrichment in tissues relevant to milk production, and any previously known association with milk production in either dairy sheep or other species.

## Discussion

The existence of a mastitis-specific ovine DNA array built on previously detected QTL regions associated with mastitis resistance in dairy sheep opens up opportunities for targeted genomic and marker-assisted selection aiming to enhance animal resistance to the disease. The aim of the present study was to investigate the association of this array with milk yield of dairy sheep and assess the feasibility of a concomitant genetic improvement programme for the two traits.

According to our results, milk yield and mastitis traits in the Chios sheep are not genetically correlated to each other. Genetic correlation estimates between milk somatic cell count and milk yield are reportedly antagonistic in dairy cattle [42] but inconsistent amongst previous sheep studies ranging from antagonistic [43] to favourable [3]. Here we considered more than 600 carefully monitored and densely phenotyped individual animals, and more than 38,000 pedigrees. We believe the genetic correlation estimates derived, ranging from -0.09 to -0.12 (Table 2), are unbiased. Even if we had a larger dataset available, rendering the standard errors small enough to qualify these estimates as significant, the practical implications would not really change; estimates would still demonstrate a very weak connection between traits. Indeed, an absolute correlation of 0.09–0.12 suggests that a very small proportion of the variation in two traits is common. Therefore, our findings indicate that selection for enhanced mastitis resistance could be incorporated into the current genetic improvement programme of the Chios sheep without incurring adverse effects on milk yield.

An overall moderate but significant heritability for milk yield was estimated in Chios sheep, consistent with the dairy sheep literature (ranging from 0.16 to 0.30) as reviewed in [44] and previous studies in Chios sheep ranging from 0.21 to 0.29 [45].

Targeted genomic analyses were conducted to further investigate the underlying correlation between milk yield and mastitis, in the context of utilising a mastitis-specific DNA array in genomic selection aiming to improve mastitis resistance. These analyses revealed several SNPs on the mastitis array with a significant effect on milk yield. However, these milk-associated SNPs were not overlapping or being in LD with genomic regions that had been previously found to affect mastitis resistance in the same population [10]. For example, the QTL for milk yield on chromosome 2 was 75 Mb distant from the one previously identified for mastitis resistance on the same chromosome [10]. The association of this QTL region with milk yield is supported by results of a previous genomic selection mapping study that compared dairy with meat sheep breeds to identify genomic regions for milk traits under selection [46]. In that study a highly homozygous region was detected in both Chios and Churra sheep in close proximity with our QTL region on chromosome 2 [46]. Furthermore, the QTL for milk yield on chromosome 12 identified in the present study was located within a previously identified QTL region for milk yield in East Friesian X Dorset cross sheep [47]. The QTLs on chromosomes 16 and 19 identified in the present study were also independent from those previously identified for mastitis resistance on the same chromosomes in the Chios sheep; the latter were located 2–4 Mb away and were in zero LD with the milk-associated region of the present study. QTLs for milk yield, milk protein and fat content have also been identified on chromosome 16 in Churra sheep [48], in close proximity with the QTL identified here in Chios sheep. To the best of our knowledge, the QTL on chromosome 19 is reported here for the first time.

In the QTL region identified for milk yield on chromosome 19 *DNAJA1* was identified as a good candidate gene. In the previous milk transcriptome study of the Churra and Assaf breeds, two other genes belonging to the same gene family, *DNAJA4* and *DNAJB2*, were reported as functional candidates for milk yield [49]. The *DNAJ* family of proteins regulate ATP hydrolysis activity, and facilitate protein folding, trafficking, prevention of aggregation and proteolytic degradation; *DNAJA1* functions as a co-chaperone and protects cells against apoptosis in response to cellular stress [50]. Therefore, this gene might affect milk yield through both metabolism and mammary apoptosis; the latter has been associated negatively with lactation persistency (daily milk yield decline in late lactation stages) in dairy species [51].

Some of the candidate genes for milk yield identified in the present study have been previously reported in dairy cattle. For example, 3-oxoacid CoA transferase 1 *(OXCT1*) has been associated favourably with both milk production [52] and mastitis resistance [53], and has been suggested to regulate mammary gland metabolism and milk synthesis during mastitis infection [54]. In our study, *OXCT1* was found to be expressed in both mammary gland and immune tissues, and highly expressed in the kidney cortex indicating that it may play a similar role in sheep. Growth hormone receptor (*GHR*) has been previously associated with increased milk yield and reduced milk somatic cell count in several dairy cattle studies [54–58]. Selective sweeps were also identified in the *GHR* region after comparing dairy and beef cattle [59]. In the present study, *GHR* was expressed in the mammary gland and the milk transcriptome, and was highly expressed in liver, relative to the other tissues sampled for the sheep gene expression atlas (http://biogps.org/sheepatlas). However, further studies, preferably including animals of the Chios breed, are needed to confirm the relevance of these genes with the regulation of milk production.

The significant SNP markers identified for milk yield in our study are mostly located in QTLs that overlap with previously identified QTLs for milk yield in other dairy sheep populations [55–57]. The only QTL reported here for the first time is on chromosome 19, which attained the highest significance level in the present study. These results are also consistent with a previous study of Chios sheep [60], suggesting that a relatively major locus might be involved in ovine milk production. Nevertheless, these SNPs were not associated with any of the studied mastitis traits.

## Conclusions

Results of the present study suggest that genetic selection for enhanced host resistance to mastitis will not antagonise milk yield in Chios sheep. Therefore, a genetic improvement programme for enhancing both mastitis resistance and milk production is feasible for this breed. In addition, there are QTLs within the mastitis specific DNA array that may be used to further increase milk production with genomic selection. Genes within genomic regions associated with ovine milk production exhibited tissue-specific expression patterns and pathways similar to those observed in cattle indicating that the underlying genetic mechanisms are likely to be, at least partially, conserved between the two species. These genes are suitable candidates for further investigation to determine if they can be exploited in breeding programmes for concomitant improvement of milk production and mastitis resistance. Admittedly, the detection of QTLs for milk yield was performed using a targeted SNP panel and not a genome-wide array; therefore our scan was very focussed and major loci associated with milk production in Chios sheep might not have been detected. Further studies using genome-wide DNA arrays are needed to identify novel QTLs for milk yield.

## Supporting information

S1 Table Linkage disequilibrium (LD) estimates (expressed as r ^2^) for the significant SNP markers identified in the genomic association analyses of milk yield and mastitis resistance in Chios sheep. (XLSX)

S2 Table Genes located in the candidate genomic regions identified for milk yield in Chios sheep. (XLSX)

S3 Table Allelic expression imbalance analysis using the one-sampled MBASED method. (XLSX)

S4 Table Selected candidate genes for milk yield in Chios sheep. (XLSX)

S1 Fig Expression level of genes located in the milk yield candidate regions, as extracted from the Churra and Assaf sheep milk transcriptome analysis. Expression level is estimated as the mean number of transcripts per million of all (5) experimental replicates and is represented here as a z-score per individual animal.
(TIF)

S2 Fig Expression level of genes located in the milk yield candidate regions, across all cell lines/tissues. Expression level is estimated as the mean number of transcripts per million (TPM) of all five (5) experimental replicates and is represented here as a z-score per cell line/tissue. Data is obtained from the sheep gene expression atlas which includes data from Texel X Scottish Blackface and Texel sheep.
(TIF)

S3 Fig Expression level of genes, located in the milk yield candidate regions, across both mammary gland and immune cell lines/tissues. Expression level is estimated as the mean number of transcripts per million of all five (5) experimental replicates and is represented here as a z-score per cell line/tissue. Data is obtained from the sheep gene expression atlas which includes data from Texel X Scottish Blackface and Texel sheep.
(TIF)
