# Supplementary figures and images for: Genetic and genomic analyses underpin the feasibility of concomitant genetic improvement of milk yield and mastitis resistance in dairy sheep
Source: PLoS One. 2019 Nov 25;14(11):e0214346. doi: 10.1371/journal.pone.0214346 (PMC6876840; doi:10.1371/journal.pone.0214346)

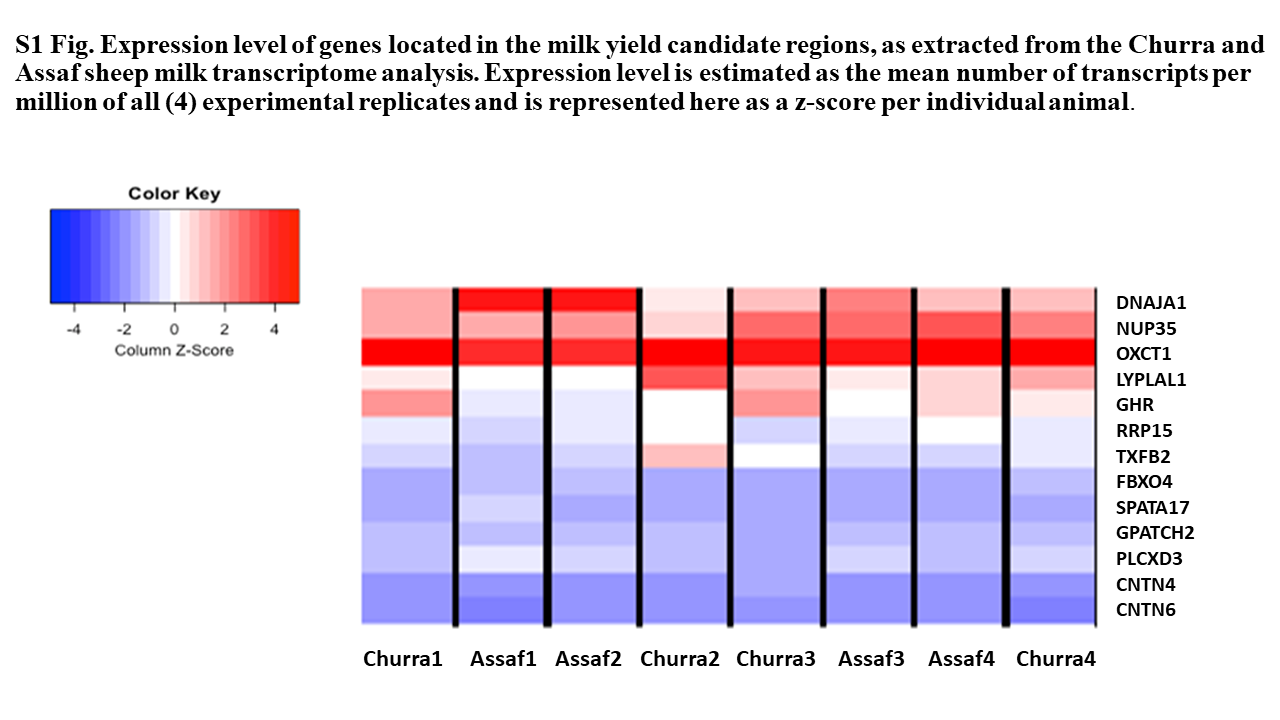

Supplement: S1 Fig — Expression level is estimated as the mean number of transcripts per million of all (5) experimental replicates and is represented here as a z-score per individual animal. (TIF) [file pone.0214346.s005.TIF]

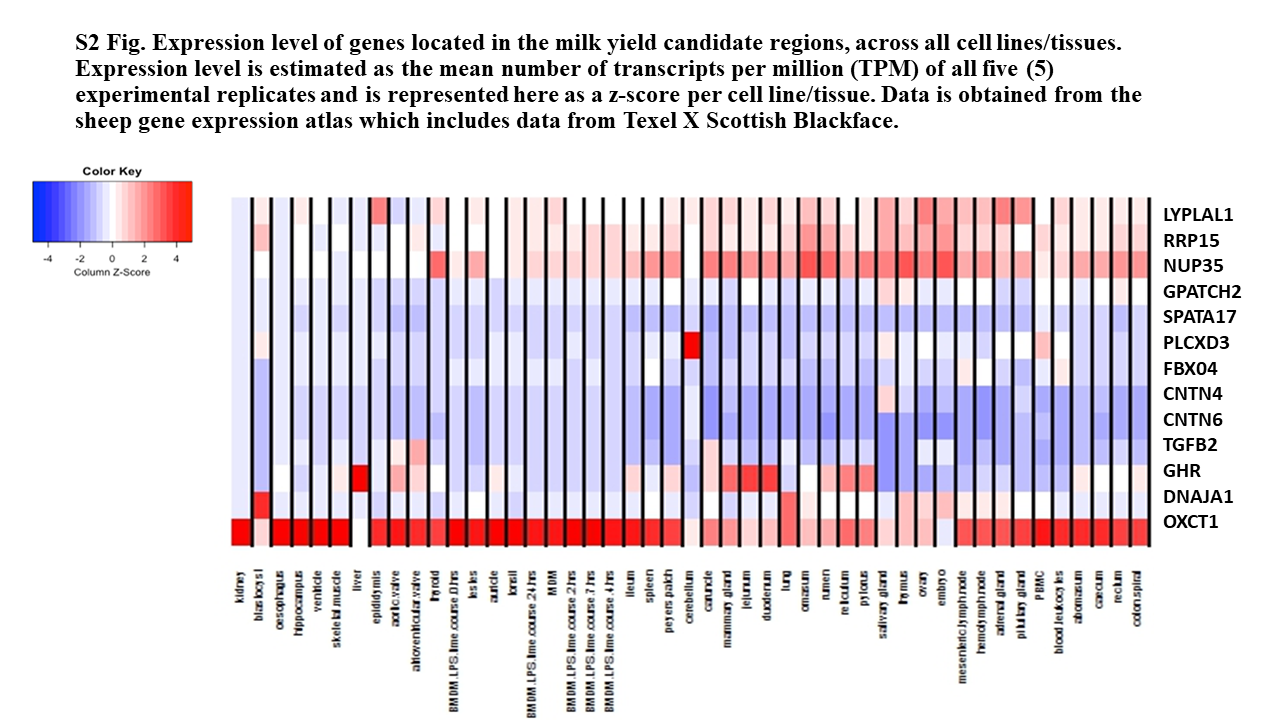

Supplement: S2 Fig — Expression level is estimated as the mean number of transcripts per million (TPM) of all five (5) experimental replicates and is represented here as a z-score per cell line/tissue. Data is obtained from the sheep gene expression atlas which includes data from Texel X Scottish Blackface and Texel sheep. (TIF) [file pone.0214346.s006.TIF]

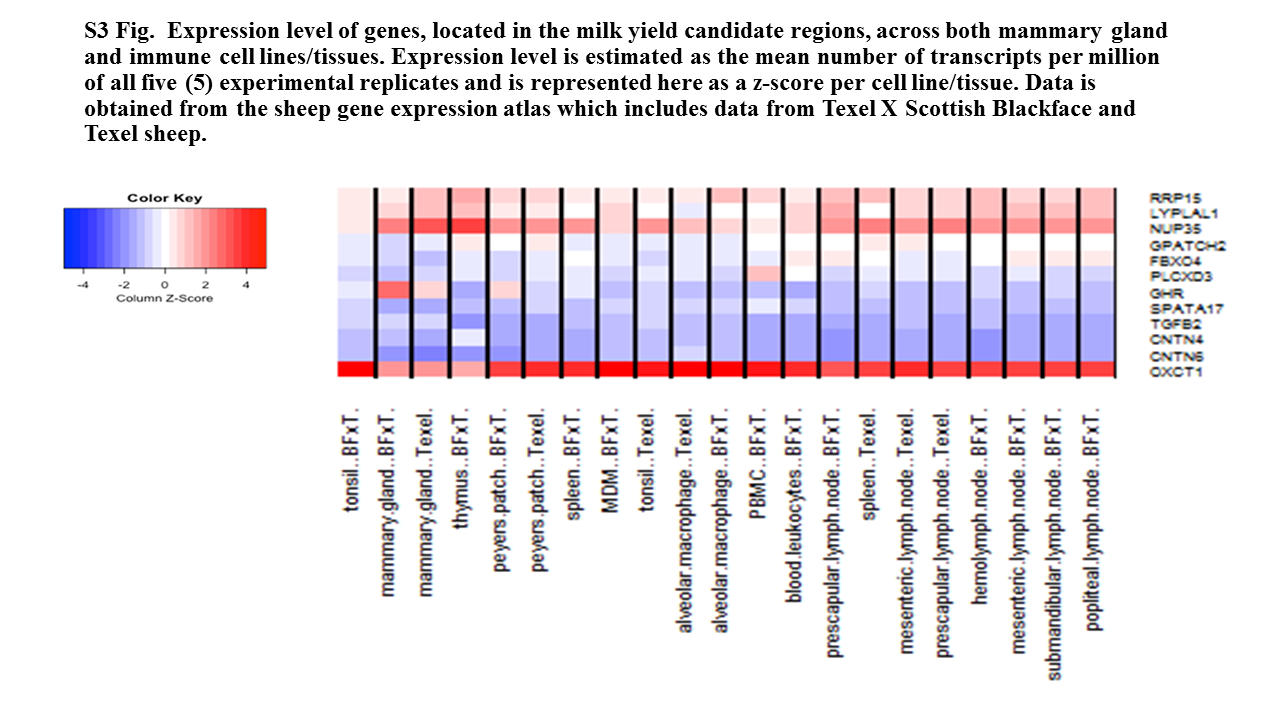

Supplement: S3 Fig — Expression level is estimated as the mean number of transcripts per million of all five (5) experimental replicates and is represented here as a z-score per cell line/tissue. Data is obtained from the sheep gene expression atlas which includes data from Texel X Scottish Blackface and Texel sheep. (TIF) [file pone.0214346.s007.TIF]
